# Evaluating Np(v) disproportionation: effect of Np concentration†

**DOI:** 10.1039/d5ra09910b

**Published:** 2026-03-23

**Authors:** Sara E. Gilson, Megan E. Simms, Frankie D. White, Laetitia H. Delmau, Luke R. Sadergaski

**Affiliations:** a Radioisotope Science and Technology Division, Oak Ridge National Laboratory 1 Bethel Valley Road Oak Ridge TN 37830 USA gilsonse@ornl.gov

## Abstract

The role of Np(v) concentration as a potential chemical driver of Np(v) disproportionation was investigated by probing Np redox chemistry in two different acid media using visible-near-infrared absorbance spectroscopy. *In situ* spectroscopy of chemically and electrochemically manipulated Np oxidation states at molar concentrations of Np underscores the difficulty in stabilizing pure Np(v), even in dilute acid. A Np(vi) component coexisting with the majority Np(v) component was observed in both HNO_3_ and HCl. Electrochemical tuning and subsequent spectroscopic analysis of the Np(VI/V) ratio revealed neptunyl self-complexation as a key mechanism occurring in both acid media. Chemical reduction of the Np(vi) component and stabilization of a minor Np(iv) component in HNO_3_ were observed; however, spectral data indicated that these two oxidation states were not observed to coexist, and pure Np(v) was stabilized only briefly. These findings demonstrate that high Np concentrations significantly affect the redox chemistry of Np in acidic solutions. Finally, the occurrence of Np(v) disproportionation could not be definitely determined by spectral data, but other mechanisms such as self-complexation and radiolysis, which potentially compete with disproportionation, must be considered.

## Abstract 2

The role of Np(v) concentration as a potential chemical driver of Np(v) disproportionation was investigated by probing Np redox chemistry in two different acid media using visible-near-infrared absorbance spectroscopy.

## Graphical abstract

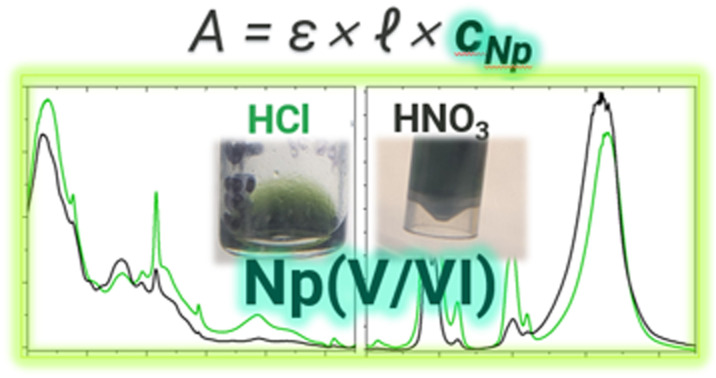

## 1. Introduction

Although Np is a significant long-term dose contributor in spent nuclear fuel and is used as target material for generating ^238^Pu used in radioisotope thermoelectric generators (RTGs), the fundamental chemistry of Np lags behind the rest of the periodic table.^1–3^ Np is most stable in the pentavalent oxidation state; however, it exhibits exceptionally complex redox chemistry and chemical behavior, and controlling these still proves challenging. The difficulty in precisely controlling Np oxidation state is caused by its redox behavior depending on multiple factors such as acid concentration, the presence of complexing agents and redox reagents, atmosphere, temperature, and Np concentration.^4–6^ Furthermore, multiple oxidation states of Np can coexist in solution due to similar redox potentials.^5,7–9^

Because of its strategic availability and radioactivity, the chemical and redox effects of high Np concentrations are especially difficult to study compared to other more abundant actinide (An) elements, such as Th and U. Studies of Np are typically restricted to small quantities and low concentrations that do not exceed more than a few milligrams per milliliter.^10^ The few studies of high Np concentrations in solution focus on the coordination chemistry and complexation behavior of the linear neptunyl Np(v)O_2_^+^ cation, particularly its tendency to self-associate and form higher-order oligomers beginning at approximately 0.1 M.^11–13^ A defining feature of Np(v) chemistry is the element's propensity to form actinyl–actinyl interactions (AAI), historically called cation–cation interactions, in which an axial oxo atom of a neptunyl cation is donated to another neptunyl cation as an equatorial ligand.^5,11,12,14^ Sullivan first observed this behavior spectrophotometrically between Np(v) and U(vi) cations in solution, although the exact reason for this unusual coordination mode remains unclear.^14,15^ Previous work hypothesized that AAIs form to meet the bonding requirements of the axial, Lewis-basic oxo atoms and that these bonds can act as magnetic superexchange pathways or precursors to the disproportionation of Np(v).^16,17^ Although studies have advanced the understanding of Np coordination chemistry in high-ionic strength systems, significant knowledge gaps remain regarding the influence of other factors on Np(v) disproportionation.

Although acid concentration is a key driver of Np(v) disproportionation, chemical equilibria dictate that Np concentration is also a factor in redox behavior. For example, the tendency of Np(v) to disproportionate in moderately to highly acidic media^4,5^ is given by eqn (1):

1 2NpO_2_^+^ + 4H^+^ ↔ Np^4+^ + NpO_2_^2+^ + 2H_2_O

Np(v) is well-known to be the dominant oxidation state in dilute acidic solutions absent of redox and complexing agents, and it disproportionates into Np(iv) and Np(vi) at higher concentrations of acid, as illustrated by eqn (1).^4,5^ The rate of Np disproportionation has been studied in various media; however, the effect of high Np(v) concentration in dilute acidic solutions is poorly understood.^11–13,18–21^ The equilibrium described by eqn (1) suggests that high concentrations of Np(v) in solution should also drive disproportionation. This equilibrium, which is fundamental to Np chemistry, motivates the hypothesis that at sufficiently high concentrations of Np(v) in dilute acid solutions, disproportionation should occur.

Disproportionation is observed in other An(V) systems, such as Pu(v), Am(v), and U(v). However, the pentavalent oxidation state of Np is remarkably stable due to its resistance to hydrolysis at pH values less than seven and its tendency to undergo disproportionation only in moderate to highly acidic media.^4^ This stability difference has been attributed to different values in the half-cell potentials of U, Np, and Pu from one another for the An(V/VI) and An(IV/V) redox couples.^22^ This difference is likely a contributing factor in the reports of different mechanisms of disproportionation that depend on the identity of the An.^22–25^ For example, the disproportionation of U(v) in perchlorate media is rapid, but the extent of this reaction can be hampered by complexation with U(vi).^26,27^ For the Pu system in 0.5 M HCl, Pu(v) disproportionates to Pu(vi) and Pu(iv), but Pu(iv) is rapidly reduced by Pu(v) to Pu(iii).^28^ Furthermore, computational studies aimed at understanding the disproportionation mechanism in An(V) systems suggest that the disproportionation mechanism for Np(v) differs significantly compared with those of U(v) and Pu(v).^29^ Although it is tempting to assume that the disproportionation mechanism is similar for all An(V) ions, differences in disproportionation mechanisms and An(V) stabilities in solution underscore that no good surrogate for Np exists. Thus, investigating the Np(v) system is crucial for obtaining a better understanding of its distinct redox behavior.

To address these knowledge gaps, we investigated the ability of high Np(v) concentrations to drive disproportionation to Np(iv) and Np(vi). In this study, visible (vis)-near-infrared (NIR) absorbance spectroscopy was used to monitor the Np oxidation state and redox behavior of high Np concentrations in dilute HNO_3_ and HCl. In the context of this study, ‘high Np concentration’ signifies Np concentrations in solution that are approximately 1 M, and ‘dilute acid’ signifies HNO_3_ or HCl concentrations that are equal to or less than 1 M. By systematically perturbing these solutions through diluting, adding redox reagents, applying electrochemical potential, and changing the acid matrix, insight was gained into fundamental Np redox chemistry.

## 2. Experimental methods

### 2.1. Caution

^237^Np (*t*_1/2_ = 2.14 × 10^6^ years) is an alpha-emitting radioisotope and poses a significant health risk. All sample preparation, measurements, and experiments were conducted in a negative-pressure glovebox in specialized facilities that are dedicated to safely handling radionuclides.

### 2.2. Chemicals and sample preparation

To evaluate Np(v) concentration as a potential driver of Np(v) disproportionation, solutions with elevated Np concentrations in dilute acid were prepared. To prevent acid-driven disproportionation and promote the stabilization of pure Np(v), samples were prepared with acid concentrations of 1 M or less. Exemplary spectra of Np(v) disproportionation at differing concentrations of HNO_3_ are provided in Fig. S1.

### 2.3. Preparation of Np in HNO_3_

Np stock solutions in HNO_3_ were prepared by weighing out solid Np nitrate crystals that were obtained in-house from the US Department of Energy's Oak Ridge National Laboratory. The solids were then dissolved in Milli-Q purity deionized water (18.2 MΩ at 25 °C) and concentrated HNO_3_ (Merck, 65% for analysis) in volumetric flasks to prepare stock solutions with the desired concentrations. After preparation, samples were given at least 24 hours to equilibrate before spectral measurements were taken. Sample compositions are listed in Table 1.

**Table 1:** Np Sample Compositions

Sample number	Np (M)	Acid (M)
1	0.89	0.8 M HNO_3_
2	1.0	1 M HCl

### 2.4. Preparation of Np in HCl

For the Np sample in HCl, a 0.223 mL aliquot of 179 g L^−1^ Np in 1 M HCl stock solution was dried down to a residue. The residue was then resuspended in 0.167 mL of 1 M HCl in a 20 mL glass scintillation vial. Sample compositions are listed in Table 1.

### 2.5. Radiochemical analyses

Np stock solution compositions were measured with alpha spectroscopy (Canberra Alpha Spectrometer Model 7401) and gamma spectroscopy to assess their isotopic purity. Alpha spectroscopy confirmed the alpha purity of the Np stock solutions with less than 2% impurities. Additionally, no significant isotopic contamination was detected *via* gamma spectroscopy apart from the presence of the ^233^Pa daughter product. Potentiometric titrations were conducted to confirm [H^+^].

### 2.6. Spectroelectrochemistry

Details of the spectroelectrochemical methods and equipment have been reported in a previous study.^30^ A modified Pt electrode card (Pine Research), as shown in Fig. S2, and a SP-300 Potentiostat (BioLogic) were used to apply an electrochemical potential to the Np solutions. For these experiments, 175 µL of Np solution was pipetted into the quartz honeycomb cell with a 1 mm pathlength. This allowed for *in situ* collection of spectra as electrochemical oxidation or reduction occurred. Initially, the Np was oxidized to Np(vi) and then reduced stepwise to Np(v) by applying increasingly more reducing potentials to the solution. A majority Np(vi) solution was obtained by applying oxidizing potentials. The oxidation of Np(v) to Np(vi) was typically observed with an applied electrochemical potential of about 1.0 V (*vs.* Ag/AgCl). Decreasing the applied potential in a stepwise manner promoted reduction of Np(vi) to Np(v). Most of the Np was converted to Np(v) by applying an overpotential of −0.1 V. It is important to note that these experiments were performed to assess Np(VI/V) spectral features through *in situ* spectral measurements of oxidation and reduction occurring at the surface of the honeycomb electrode, and not to electrochemically oxidize or reduce the entire sample. After measurements, sample aliquots were stored separately from the stock solutions and not reused for experimental work.

### 2.7. Oxidation state adjustment with H_2_O_2_

For experiments that required oxidation state adjustment, 10% H_2_O_2_ was added to the sample or the stock solution to reduce the Np(vi) to Np(v) or Np(iv). A 175 µL aliquot of Np stock solution that was treated with H_2_O_2_ was pipetted into a quartz cuvette with a 1 mm pathlength and capped to prevent sample evaporation, then measured in a cuvette holder. A spectrum was collected every two minutes for approximately 48 hours. After the experiment was complete, the sample was stored separately from the stock solution and not used reused for experimental work.

### 2.8. Spectrophotometry

Spectra were collected using 1 mm path length quartz cuvettes. Ultraviolet-vis-near infrared (UV-vis-NIR) spectra were acquired using QEPro and NIRQuest (Ocean Insight) spectrophotometers. As described previously, a stabilized W-halogen lamp (ThorLabs) was used as the light source.^30^ A SL2 Mercury Argon calibration lamp (StellarNet) was used for wavelength calibration. Ultraviolet-vis-NIR spectra were collected from 320 to 1115 nm with a step size of 0.8 nm, and NIR spectra were collected from 892 to 1700 nm with a step size of 1.59 nm.^30^ Spectra were averaged accordingly to optimize the signal-to-noise ratio and were collected in triplicate unless otherwise noted. UV-vis spectra were preprocessed from 380 to 950 nm and corrected using a linear baseline correction. NIR spectra were preprocessed from 900 to 1360 nm and corrected using a linear baseline correction. No other preprocessing or data corrections were applied and spectral preprocessing was consistent for all data collected. Spectral preprocessing and baseline correction were executed using Vektor Direktor (2.0) from the KAX Group.

## 3. Results and discussion

**Fig. 1:**
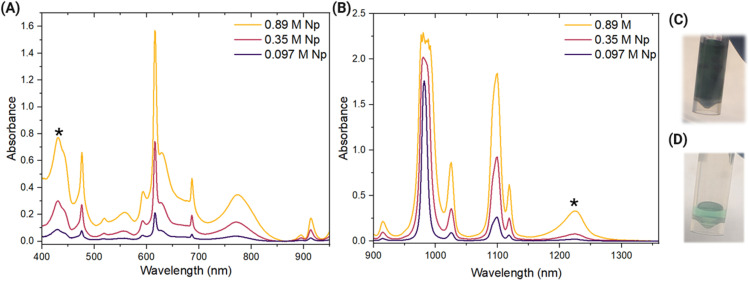
(A) Vis and (B) NIR spectra of 0.89 M, 0.35 M, and 0.097 M Np in 0.8 M HNO_3_. Features associated with Np(vi) are denoted with asterisks. Transmitted light images of the (C) 0.89 M Np and (D) 0.097 M Np.

Even in dilute HNO_3_ solution, in which Np(v) is most stable, pure Np(v) was not observed in solutions with molar Np concentrations. Instead, vis-NIR spectra of 0.89 M Np in 0.8 M HNO_3_ indicate the presence of a minor Np(vi) component at 1222 nm in addition to the majority Np(v) component, as shown in Fig. 1. Spectral features associated with Np(vi) are denoted by asterisks. As given in Table 2, the Np(vi) component constituted approximately 8% of the total Np concentration whereas the Np(v) component accounted for approximately 92% of the total Np concentration (details of Np oxidation state ratio calculations are given in the SI). Because dilute acidic media without additional redox or complexing agents typically stabilize Np(v), the presence of this Np(vi) component is somewhat unexpected.

**Table 2:** Total Np concentrations and oxidation state percentages with dilution

Total Np (M)	Percent Np(v) (%)	Percent Np(vi) (%)
0.89	91.8(1)	8.2(1)
0.35	94.7(2)	5.3(2)
0.097	100.0(5)	<0.5

The ratio of Np oxidation states in dilute HNO_3_ with high Np concentration depends on the total concentration of Np in solution. Systematically diluting the 0.89 M Np sample to approximately 0.35 M and 0.097 M Np with 0.8 M HNO_3_ to maintain acid concentration resulted in decreased intensity of all spectral features (Fig. 1). However, the ratio of Np(v) and Np(vi) oxidation states changed with each dilution; the Np(vi) component decreased and the Np(v) component increased with total Np concentration, as shown in Table 2. In the spectrum of the 0.35 M Np sample, Np(vi) was calculated as approximately 5%, which decreased from 8% in the initial sample. Dilution of the 0.89 M Np sample to 0.097 M Np yielded a spectrum with only Np(v) and no detectable Np(vi). These spectra indicate that decreasing the total Np concentration in solution altered the Np oxidation state ratios and decreased the minor Np(vi) component until it was no longer spectrophotometrically detected. Horne *et al.* also reported the dependency of the Np(vi) and Np(v) distribution ratios on gamma irradiation dose and acid concentration for gamma-irradiated solutions of Np in different concentrations of HNO_3_.^31^ However, in that study, the samples were externally irradiated using a ^60^Co source and contained millimolar Np concentrations, whereas in the current study, no external irradiation was applied to the samples, and acid concentration was maintained during sample dilution.

**Fig. 2:**
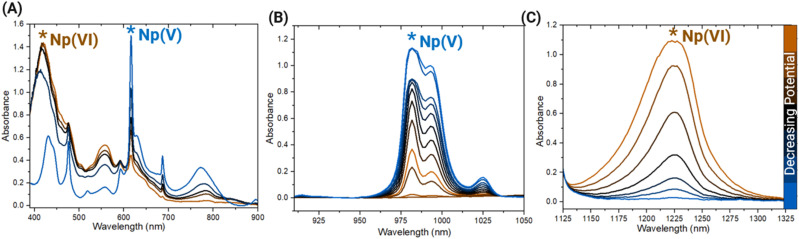
Select absorbance spectra acquired during the electrochemical reduction from Np(vi) to Np(v) in the 0.89 M Np sample in the (A) vis and NIR regions at the primary (B) Np(v) and (C) Np(vi) peaks.

Electrochemically stabilizing different ratios of Np(v) and Np(vi) in the 0.89 M Np sample in HNO_3_ revealed extensive Np self-complexation, providing further insight into Np redox behavior. After adjusting the ratio of Np(v) and Np(vi) components, the primary peaks associated with these oxidation states were probed for changes in character and position. For Np(v), the characteristic 980 nm band exhibits splitting (Fig. 2) that is not clearly resolved in the initial spectrum that was collected without applying an electrochemical potential. Two peaks were observed in this broad signal at approximately 980 and 990 nm. The initial report of AAIs in solution by Sullivan *et al.* noted splitting in the Np(v) 980 nm peak and attributed it to the interaction between Np(v)O_2_^+^ and U(vi)O_2_^2+^.^14^ Additional Raman spectroscopic studies have posited that AAIs forming between neptunyl cations can occur especially at elevated concentrations of Np, such as those of the current study.^11,12^ Recent spectroscopic studies of acidic Np solutions that were gradually concentrated through slow evaporation and laser heating also suggested that AAIs form between neptunyl ions in solution.^13,32^ The splitting in the 980 nm band observed in Fig. 2B is comparable with that reported in the literature for the Np(v) system and spectra of AAIs, indicating that AAI formation also likely occurs between neptunyl ions in the 0.89 M Np sample.^14,33–35^ Notably, the lack of discernible changes in the characteristic Np(vi) peak at 1222 nm with varying ratios of Np(v) and Np(vi) agrees with previous work. A study by Madic that titrated Np(v) into a solution of Np(vi) did not result in changes in the 1222 nm peak position, although weak complexation was postulated by calculating formation constants.^11^

**Fig. 3:**
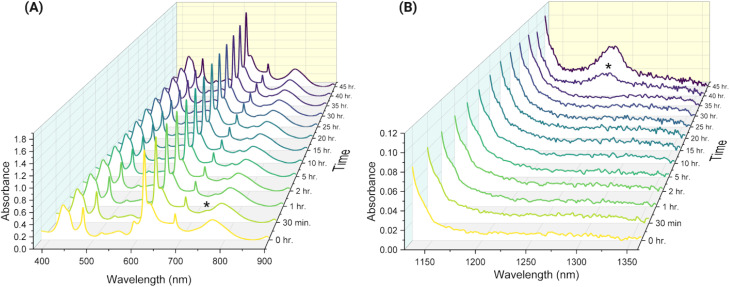
(A) Vis and (B) NIR spectra of the 0.89 M Np after addition of H_2_O_2_. Signals associated with Np(iv) in the vis region and Np(vi) in the NIR region are denoted with asterisks.

**Fig. 4:**
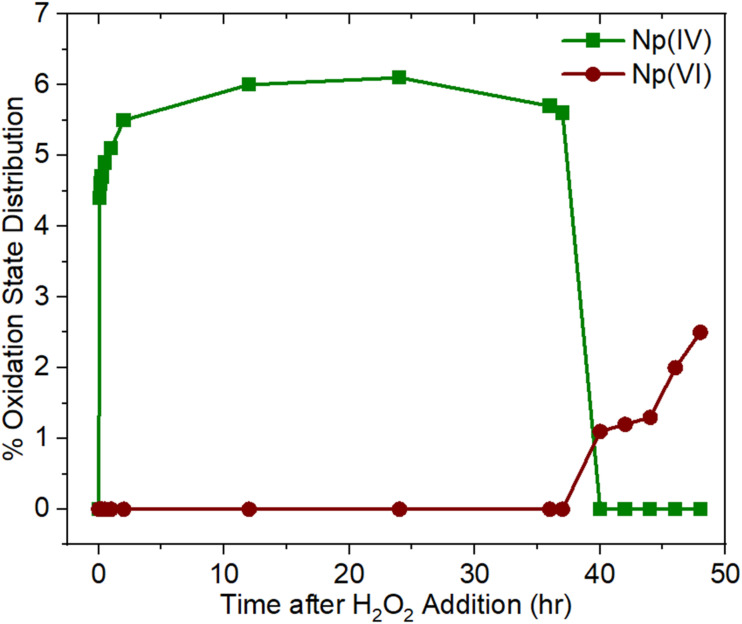
Percentage of Np(iv) and Np(vi) oxidation states in 0.8 M HNO_3_ as a function of time after H_2_O_2_ addition.

Chemical reduction of the Np(vi) component using H_2_O_2_ and monitoring *via* spectrophotometry *in situ* for approximately 48 hours demonstrated that a minor Np(iv) component is temporarily stabilized. However, these minor Np(iv) and Np(vi) components were not observed to coexist in the experimental conditions studied here. The Np(vi) component was reduced within one minute of adding H_2_O_2_, and after approximately ten minutes, a small signal at approximately 725 nm appeared, indicative of Np(iv). Spectra are shown in Fig. 3, with Np(iv) and Np(vi) features denoted by asterisks. Pure Np(v) was stabilized only briefly before the Np(iv) component grew in. The small Np(iv) component persisted for approximately 37 hours until it oxidized, and Np(vi) reappeared at 1222 nm. Interestingly, the spectral data collected continuously during this experiment indicate that the Np(iv) and Np(vi) components were not observed to coexist under the investigated experimental conditions, suggesting that their conversion is possibly related. Np mass balance calculations and Np(IV, V, and VI) ratios for specific timepoints are given in the SI. The percentages of Np(iv) and Np(vi) illustrated in Fig. 4 demonstrate that the system contains two oxidation states of Np most of the time during which data were collected, with Np(v) as the majority component. Again, a pure Np(v) spectrum was observed for less than five minutes before ingrowth of the Np(vi) component, underscoring the difficulty in stabilizing a high concentration of pure Np(v).

**Fig. 5:**
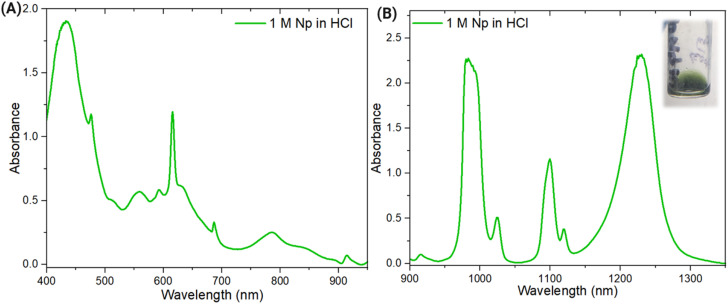
(A) Vis and (B) NIR spectra of 1 M Np in HCl with a (B, inset) transmitted light photograph of the Np in HCl solution.

Probing Np redox behavior in another acid matrix, 1 M HCl, at 1 M Np revealed the presence of both Np(v) and Np(vi), indicating that the presence of a minor Np(vi) component is not specific to the HNO_3_ medium. Although the ratio of Np(v) and Np(vi) oxidation states is different for HCl and HNO_3_, this cannot be definitively attributed to the differences in acid matrix (Fig. 5). The different oxidation state ratios could be caused by slightly different equilibration times of the samples which were prepared and measured at different times. Additionally, it is worth noting that the Np in both the HCl and the HNO_3_ samples was in secular equilibrium with the ^233^Pa daughter when these samples were prepared. The primary finding from comparing these spectra was that pure Np(v) was not observed spectrally in either dilute HNO_3_ or HCl media.

**Fig. 6:**
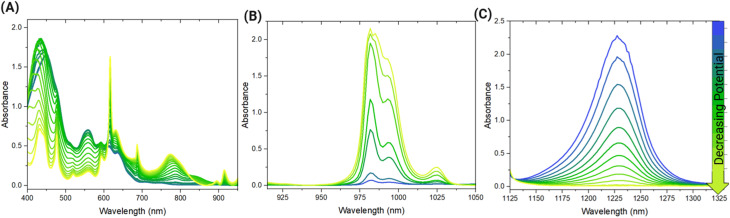
Select absorbance spectra acquired during the electrochemical reduction from Np(vi) to Np(v) in the 1 M Np in HCl sample in the (A) vis and NIR regions at the primary (B) Np(v) and (C) Np(vi) peaks.

Similar spectral features of different Np(v) and Np(vi) ratios in the HCl system indicate that Np self-complexation also occurs in the 1 M Np in HCl system. Splitting in the Np(v) 980 nm peak was observed throughout the spectroelectrochemical scan, as shown in Fig. 6. For the 1222 nm peak associated with Np(vi) shown in Fig. 6C, changes in peak shape and position are not apparent. The behavior of the Np(v) and Np(vi) signals is comparable with those of the HNO_3_ system, suggesting that Np self-complexation and AAI formation likely occur in both systems. The findings of this study underpin the complexity of Np redox behavior, especially in systems with high concentrations of Np. Despite the dominance of Np(v) in dilute acid media at millimolar concentrations, this same behavior was not observed in solutions with molar concentrations of Np.

Additionally, chemical and electrochemical manipulation of Np oxidation states in these concentrated systems demonstrated Np self-complexation and AAI formation as mechanisms of interaction occurring in solution. Even in different acid media—HNO_3_ and HCl—similar Np redox behavior was observed with the presence of both Np(v) and Np(vi). Many variables warrant further discussion in these high-concentration Np systems. The source of Np(vi) in these dilute acidic systems is not straightforward; however, extensive literature on radiolysis of HNO_3_ indicates that the presence of the Np(vi) component in this system could potentially be attributed to Np interacting with its acid matrix. Literature reports that the oxidation of Np(v) to Np(vi) in HNO_3_ can occur because of the presence of HNO_2_.^36,37^ More specifically, Tochiyama *et al.* suggest that the ˙NO_2_ radical is the oxidizing agent of Np(v) that leads to the formation of Np(vi), as shown in eqn (2):^36^

2 NpO_2_^+^ + 3/2H^+^ + 1/2NO_3_^−^ ↔ NpO_2_^2+^ + 1/2HNO_2_ + 1/2H_2_O

Additional work by Mincher *et al.* and Horne *et al.* expanded on this empirical description and identified the hydroxyl radical (OH˙) and nitrate radical (NO_3_˙) as products of HNO_3_ radiolysis that can oxidize Np(v) to form Np(vi). These studies also found that the radiolytic yield of these oxidizing radicals affects the ratio of these two oxidation states in solution.^31,37^

However, the presence of Np(v) and Np(vi) in both HNO_3_ and HCl suggests that the Np(vi) component could form due to another mechanism that is distinct from N-containing oxidizing radicals generated from HNO_3_. Because of the rather dilute concentration of the HCl (1 M), water radiolysis products could also possibly oxidize Np(v) to Np(vi) in this system.^38^ The OH˙ radical is a highly oxidizing product of water radiolysis and is known to alter Pu oxidation state ratios in solution.^38,39^ H_2_O_2_ is another product of water radiolysis that can alter Np redox behavior; however, its redox chemistry with Np is highly dependent on solution conditions and is not straightforward.^40^ These or other species may possibly cause oxidation to Np(vi) in the HCl system, but further experimental work and multiscale modeling are needed to confirm this hypothesis.

The proposed reasoning for the presence of the Np(vi) component in both HNO_3_ and HCl is that the reaction requires conditions that promote solution radiolysis. As mentioned previously, the samples in this study were not subjected to external radiation, so radiolysis would have to occur through radioactive decay of Np and its daughter products. ^237^Np is an alpha-emitting radioisotope with an alpha particle decay energy of approximately 4.8 MeV, and its daughter product ^233^Pa decays through beta and gamma emissions of 0.568 and 0.312 MeV, respectively.^4,41^ The radioactive decay and radiolytic impact of Np on most lab-scale systems are usually neglected because of Np's long half-life and the small quantities that are typically used.^40^ However, the systems investigated in this study contained molar concentrations of Np; thus, the radiolysis effects on the system from both Np and Pa possibly were no longer negligible and did affect the oxidation state of Np.^40^ To estimate these radiolysis effects, the total energy from the decay of ^237^Np and ^233^Pa deposited in 1 mL of a 0.8 M Np solution was approximated using decay energies from Chadwick *et al.*^42^ For these calculations, Pa was assumed to be in secular equilibrium with the Np. The total energy deposited in solution was estimated to be approximately 3.71 × 10^−2^ Gy per s per mL, corresponding to 3.21 kGy per mL in 24 hours (details of the calculation are given in the SI). In comparison, Np-HNO_3_ solutions that undergo external irradiation from a ^60^Co source typically accumulate doses in the kGy regime before subsequent investigation.^31^ It is possible that this quantity of energy deposited from the decay of Np and Pa could potentially cause radiolysis effects of the solvent that lead to Np(vi) formation, however, further studies are needed to confirm this hypothesis.

Although spectral data suggest the occurrence of Np self-complexation in both HNO_3_ and HCl, they do not directly support the occurrence of Np(v) disproportionation, which was the primary motivation behind this work. The Np(vi) coexists with Np(v) in both HNO_3_ and HCl, however no Np(iv) component was detected in this study spectroscopically except when a redox reagent (H_2_O_2_) was added to the system. According to the chemical equilibrium described in eqn (1), Np(v) disproportionation should result in equal molar concentrations of Np(iv) and Np(vi). Thus, if the Np(vi) component in this system forms from disproportionation, an equal molar concentration of the Np(iv) component should also form. Furthermore, because the Np(vi) NIR peak at 1222 nm and the series of Np(iv) peaks in the vis region have comparable molar extinction coefficient values in dilute HNO_3_, if disproportionation occurs and Np(iv) forms and persists, Np(iv) should be spectrophotometrically detectable along with the Np(vi) component.^30,43^

Notably, Np(iv) is not inherently stable in the dilute acidic conditions that were used to potentially promote Np(v) disproportionation in this study. If disproportionation occurs, the Np(iv) could be rapidly destabilized due to these unfavorable solution conditions, which has been observed for certain Pu oxidation states, such as Pu(v), in HNO_3_.^44^ Compared with Np(v) and Np(vi), Np(iv) is relatively unstable in dilute acidic conditions unless a redox reagent, complexing ligand, or electrochemical potential (*E*_0_ NpO_2_^+^/Np^4+^ = 0.07 V *vs.* Ag/AgCl in 1.01 M HNO_3_) is applied.^4,5,9^ However, as mentioned previously, the occurrence of disproportionation in these systems cannot be concluded from the current spectral data.

The findings of the spectral data do not clearly indicate the occurrence of Np(v) disproportionation, and multiple explanations are possible for why disproportionation may not occur in the studied systems. The equilibrium conditions for disproportionation described in eqn (1) may not be fulfilled—specifically, acid concentration, which is fourth-order in the equilibrium expression for this reaction. Without sufficient [H^+^] in the system, perhaps Np(iv) cannot form even with an excess of Np(v) in the system. It is also feasible that Np self-complexation and the formation of AAIs, as indicated by spectral data, inhibit disproportionation from occurring. Although previous work has suggested that AAIs can enhance disproportionation, further studies are needed to progress our understanding of the relationship between AAIs and disproportionation.^16,17^ Finally, radiolysis effects on the system from the high concentration of Np and the Pa daughter possibly outcompete disproportionation, resulting in the formation of a minor Np(vi) component. Other explanations are also possible, and further study, especially of the effect of radiolysis products on Np redox chemistry, is needed to gain insight into the interplay of variables in these systems. Computational efforts that investigate the kinetic and thermodynamic stability of Np(iv) in these conditions would be beneficial for informing future experimental work. Future studies should determine if an achievable set of tailored solution conditions in dilute acid can be derived from thermodynamic equilibria and pursued experimentally.

## 4. Conclusions

Spectral data from this study underscore the complexity of Np redox chemistry, as illustrated by the difficulty in stabilizing pure Np(v) at high concentrations even in dilute acidic media. The effect of Np concentration as a driver of Np(v) disproportionation in dilute acidic media was examined. Attempts to stabilize sufficient concentrations of Np(v) to potentially promote disproportionation led to insights into the chemical behavior of the minor Np(vi) component that coexists with Np(v) at high Np concentrations. The presence of this minor component in both HNO_3_ and HCl matrices highlights the importance of considering radiolysis effects of radioisotopes that are typically neglected in lab-scale studies when small quantities are used.

Additionally, the complexation occurring in these high-concentration Np systems highlights the need to develop an understanding of the relationship between AAIs and Np(v) disproportionation. Enhanced knowledge of these two mechanisms would enable strategies for better Np redox control and advance fundamental knowledge of this element. Future studies should focus on tailoring solution conditions that could potentially promote Np(v) disproportionation as well as complexation to gain insight into the chemical behavior of this oxidation state.

The findings of the current study emphasize the need for additional studies of high-ionic strength systems with high concentrations of An to better understand how elevated concentrations of An radiolytically and chemically affect their local and extended systems. Developing this knowledge would lead to advancements in the fundamental and applied fields of An science.

## Author contributions

The manuscript was written through contributions of all authors. All authors have given approval to the final version of the manuscript. S. Gilson: conceptualization, methodology, investigation, formal analysis, data curation, visualization, writing – original draft, review, and editing. M. Simms: investigation, review, and editing. F. White: investigation, review, and editing. L. Delmau: conceptualization, methodology, review, and editing. L. Sadergaski: conceptualization, methodology, review, and editing.

## Conflicts of interest

The authors have no competing financial interests to declare.

## Supplementary Material

RA-016-D5RA09910B-s001

## Data Availability

The datasets that were generated and analyzed as part of current study are available from the corresponding author upon reasonable request. Supplementary information (SI): additional spectra and tables. See DOI: https://doi.org/10.1039/d5ra09910b.
