# Supplementary material for: Evaluating Np(v) disproportionation: effect of Np concentration
Source: RSC Adv. 2026 Mar 23;16(18):16040–9. doi: 10.1039/d5ra09910b (PMC13007889; doi:10.1039/d5ra09910b)
Supplement: RA-016-D5RA09910B-s001 [file RA-016-D5RA09910B-s001.pdf]

Supplementary Information (SI) for RSC Advances.
This journal is © The Royal Society of Chemistry 2026

Supporting Information for:

Evaluating Np(V) Disproportionation: Effect of Np Concentration

Sara E. Gilson, Megan E. Simms, Frankie D. White, Laetitia H. Delmau, Luke R. Sadergaski*

Radioisotope Science and Technology Division, Oak Ridge National Laboratory, 1 Bethel Valley Road, Oak Ridge, TN 37831, USA

*Corresponding author: gilsonse@ornl.gov

This supporting information contains:

Figures: 2

Tables: 2

Pages: 5

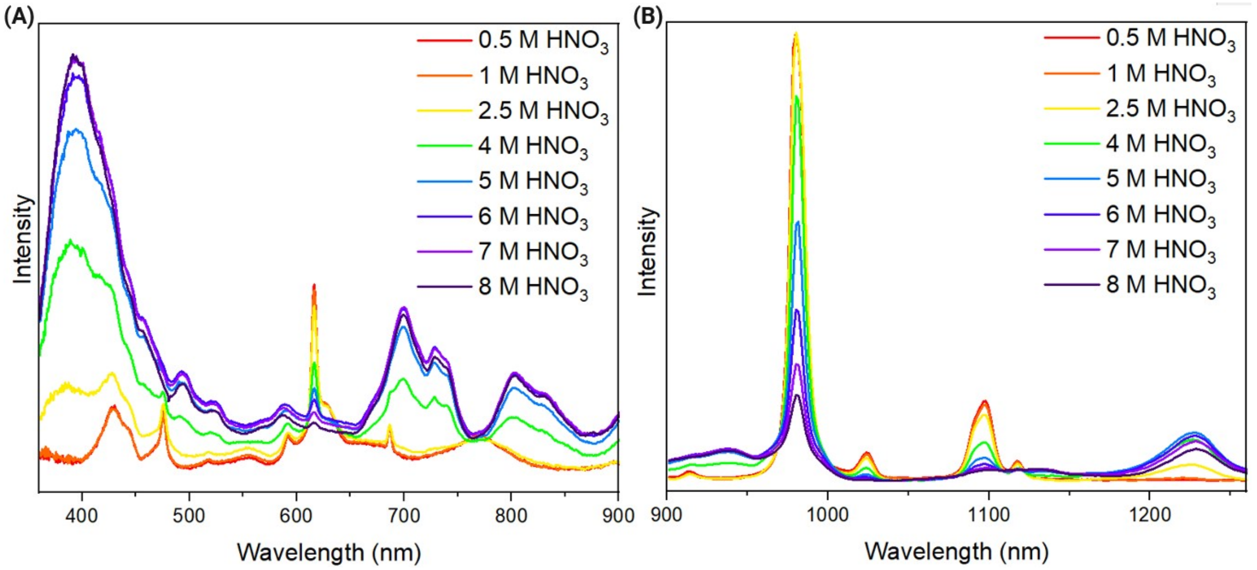

Figure S1. UV-Vis (A) and NIR (B) spectra of approximately 0.08 M Np solutions in various concentrations of HNO_3, exhibiting different degrees of disproportionation.

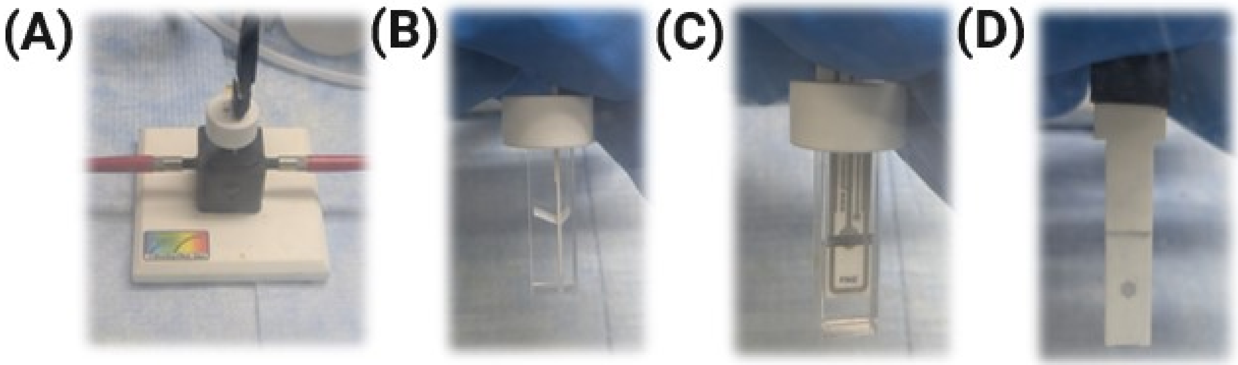

Figure S2. Images of (A) the spectropotentiometric setup, (B, C) the side and front of the modified honeycomb electrode in the 1 mm pathlength cell, and (D) the back of the modified honeycomb electrode.

Np Oxidation State Ratio Calculations

Np oxidation states were described as ratios based on Np concentrations that were calculated from baseline corrected absorbance spectra. Molar extinction coefficients for characteristic peaks Np(V) over a range of Np concentrations have been evaluated extensively and found to not behave linearly at molar concentrations of Np above 0.45 M, with details given by Sadergaski et al.[1] This work extensively tested the behavior of solutions with different concentrations of Np with cuvettes of different pathlengths and calculated molar extinction coefficients for quantification while accounting for distortions and changes in Np absorption bands due to complexation and other high concentration effects.[1] These molar extinction coefficients were used in the quantification of Np(V) in the current study and are given below in Table S1. Np(VI) molar extinction coefficients were also studied extensively at different acidities and the value of the coefficient for the characteristic Np(VI) peak at 1222 nm in 1 M HNO_3 was determined to be 42 $M^{-1}cm^{-1}$.[2] For Np(IV) quantification, the molar extinction coefficient 54 $M^{-1}cm^{-1}$ for the 724 nm peak was used for quantification.[3] Concentrations of the different Np oxidation states were determined using Beer's Law and characteristic peaks of each Np oxidation state that do not overlap significantly with other spectral signals that could interfere and lead to overestimated Np concentrations. Np(V) and Np(VI) concentrations were calculated using the 617 nm and 1222 nm peaks, respectively. Np(IV) concentrations from the H_2O_2 study were quantified using the 723 nm peak. Total Np concentrations were determined by adding the concentrations of all Np oxidation states that were observed in a given spectrum. Ratios of each Np oxidation state were then calculated by dividing the concentration of the given Np oxidation state by the total Np concentration and then multiplying by a factor of 100.

Analysis of representative baseline spectra and instrument response indicate that the minimum optical density that can be detected is 0.02 absorbance units. This value, in combination with the molar extinction coefficients for Np(VI) and Np(IV) peaks given above and the 1 mm pathlength used in the experiments, provides an approximate limit of detection of 4.8 mM Np(VI) and 3.7 mM Np(IV), which are approximately 0.5% of the total 0.89 M Np concentration in this study.

Table S1. Molar Extinction Coefficients Used for Np(V) Quantification[1]

Np Concentration (M)	Molar Extinction Coefficient of Np(V) 617 nm Peak ($M^{-1}cm^{-1}$)
0.886	17.6
0.440	22.2
0.089	22.3

Table S2. Mass Balance of Np and Oxidation States Ratios in H_2O_2 Time Experiment

Time After H_2O_2 Addition	Np(IV) (M)	Np(V) (M)	Np(VI) (M)	Total Np (M) (0.89 M by alpha spectroscopy)
5 minutes	0.034	0.85	0.00	0.88
10 minutes	0.034	0.85	0.00	0.88

15 minutes	0.036	0.84	0.00	0.88
30 minutes	0.037	0.84	0.00	0.88
1 hour	0.039	0.84	0.00	0.88
2 hours	0.042	0.84	0.00	0.88
12 hours	0.045	0.83	0.00	0.88
24 hours	0.046	0.85	0.00	0.90
36 hours	0.044	0.86	0.00	0.90
37 hours	0.044	0.86	0.00	0.90
40 hours	0.00	0.86	0.032	0.89

Calculations of Total Energy Deposited in Solution from ^{237}Np and ^{233}Pa Decay

To estimate the energy deposited in a 1 mL solution of 0.8 M (211 g/L) Np in a dilute nitric acid solution being in secular equilibrium with its daughter Pa-233, the Q-Values for the decay of each radionuclide were used. The Q-value is the energy released in the reaction or decay.

For Np-237, the alpha energy was assumed to be totally absorbed into the material, considering the short range of alpha particles in material. The Q-value for Np-237 alpha decay given by the BNL National Nuclear Data Center (https://www.nndc.bnl.gov/nudat3/mird/) is 4.973 MeV per alpha decay with a probability of 100%.[4] The activity of the given solution is 3.29E8 dpm/ml (5.48E6 dps/ml), and the energy released and absorbed is 4.973 MeV per disintegration (2.73E7 MeV/sec or 4.37E-6 Joules/sec). Since 1 Gray is 1 Joule/kg and assuming a time period of one second and a volume of one mL, a density of the solution of 1.311 g/ml (1.1 g/ml HNO_3 (estimated) + 0.211 g/ml Np-237) gives:

((4.37E-6 Joules/sec)/ (1.311g)) (1000 g/kg) = <u>3.33 E-3 Gray/sec/mL</u>

For Pa-233, it was assumed that Pa-233 in solution is in secular equilibrium with Np-237. To obtain the upper limit of energy absorbed, it was assumed that 100% of the beta energy is totally absorbed into the solution. The daughter of Pa-233 (U-233) has a much greater half-life than Pa-233 and its activity is 5 orders of magnitude less than Pa-233, and was therefore ignored in this calculation. The Q-value for Pa-233 alpha decay given by the BNL National Nuclear Data Center (https://www.nndc.bnl.gov/nudat3/mird/) is 0.5703 MeV per beta decay with a probability of 100%.[4] The activity of the given solution is 3.29E8 dpm/mL (5.48E6 dps/mL), and the energy released and absorbed is 0.5703 MeV per disintegration, or 3.13E6 MeV/sec. Converting MeV/sec to Joules/sec gives 5.01E-7 Joules/sec (using 1.6022E-13 Joules/MeV). Since 1 Gray is 1 Joule/kg and assuming a time period of one second, a volume of one mL, a density of the solution of 1.311 g/ml (1.1 g/ml HNO_3 (estimated) + 0.211 g/ml Np-237) gives:

((5.01E-7 Joules/sec)/ (1.311g)) (1000 g/kg) = <u>3.82 E-4 Gray/sec/mL</u>

Total energy deposited per sec: <u>3.71E-2 Gray/sec/mL</u>

References:

(1) Sadergaski, L. R.; Patton, K. K.; Toney, G. K.; DePaoli, D. W.; Delmau, L. H. *Measuring Neptunium Concentration using Optical Spectrometry for the Pu-238 Supply Program*; ORNL/TM-2021/2072; Oak Ridge National Laboratory (ORNL), Oak Ridge, TN (United States), United States, 2021. https://www.osti.gov/biblio/1826033

https://www.osti.gov/servlets/purl/1826033DOI: 10.2172/1826033.

(2) Gilson, S. E.; Sadergaski, L. R.; Parkison, A. J. Investigating Np(VI) Nitrate Speciation Control through Temperature and Optical Spectroscopy. *Acs Omega* **2025**. DOI: 10.1021/acsomega.5c05359.

(3) Gilson, S. E.; Andrews, H. B.; Sadergaski, L. R.; Parkison, A. J. Insights into Tetravalent Np Speciation in HNO3 through Spectroelectrochemistry and Multivariate Analysis. *Acs Omega* **2024**. DOI: 10.1021/acsomega.4c05464.

(4) Chadwick, M. B.; Herman, M.; Obložinský, P.; Dunn, M. E.; Danon, Y.; Kahler, A. C.; Smith, D. L.; Pritychenko, B.; Arbanas, G.; Arcilla, R.; et al. ENDF/B-VII.1 Nuclear Data for Science and Technology: Cross Sections, Covariances, Fission Product Yields and Decay Data. *Nuclear Data Sheets* **2011**, *112* (12), 2887-2996. DOI: https://doi.org/10.1016/j.nds.2011.11.002.
